# Remote sensing reveals that wild herbivores limit senescent vegetation accumulation on dryland conservation reserves

**DOI:** 10.1002/eap.70239

**Published:** 2026-04-20

**Authors:** Matt S. Smith, Adrian G. Fisher, Graeme Finlayson, Mike Letnic

**Affiliations:** ^1^ School of BEES, UNSW Sydney New South Wales Australia; ^2^ Bush Heritage Australia Melbourne Victoria Australia; ^3^ Department of Ecology and Evolutionary Biology The University of Adelaide Adelaide South Australia Australia

**Keywords:** arid, conservation land management, grazing impacts, herbivores, rainfall gradient, resource pulse, satellite imagery, Sentinel‐2

## Abstract

Wild herbivores threaten vegetation recovery on dryland conservation reserves globally. Monitoring herbivore impacts in remote drylands is difficult because vegetation biomass transitions between living and senescent states in response to irregular precipitation events. However, land managers need detailed understanding of the impacts that wild herbivores have on vegetation to develop and refine herbivore management strategies. Remote sensing provides the ability to assess grazing impacts on living and senescent vegetation with high temporal and spatial resolution. Here, we use Sentinel‐2 satellite imagery to investigate how grazing by kangaroos and rabbits impacted fractional cover of photosynthetic (PV) and non‐photosynthetic (NPV) vegetation over a 7‐year period on three dryland reserves with experimental herbivore exclusion plots. We compared PV and NPV cover in plots that were accessible to all herbivores, accessible to kangaroos only, and inaccessible to both rabbits and kangaroos. Generalized linear mixed models were used to determine if the grazing impacts of rabbits and kangaroos varied from each other, between reserves, and in response to variable rainfall patterns. Grazing impacts varied between each herbivore, conservation reserve, and between PV and NPV. PV was only weakly limited by kangaroos across all reserves and antecedent rainfall totals. NPV was limited by rabbits and kangaroos, with grazing having stronger impacts on NPV than PV. The grazing impacts of rabbits and kangaroos varied spatially with evidence that NPV was limited by kangaroos only, by rabbits only, and by both species across different reserves. Both herbivores had stronger impacts on NPV as antecedent rainfall decreased. Our results show that the impact of herbivores on vegetation biomass is greatest during periods of dry climatic conditions. These findings contribute to a growing body of evidence showing that grazing by wild herbivores can have detrimental impacts on dryland ecosystems by disrupting ecological processes supported by NPV. Our results highlight the importance of herbivore management during productive periods to ensure NPV is retained during periods of low rainfall.

## INTRODUCTION

Only 12% of the world's drylands are protected within conservation reserves (Lewin et al., 2024). Efforts to restore vegetation on these reserves can be hindered where populations of wild herbivores persist at unsustainable levels (Mills et al., 2020). Data addressing when and how wild herbivores jeopardize vegetation recovery would allow land managers to develop and refine effective herbivore control programs (Gordon et al., 2004). However, investigating grazing effects in dryland systems can be difficult because (1) remote dryland areas are often difficult to access leading to intermittent in situ data collection (Verstraete et al., 2011) and (2) dryland vegetation can be highly dynamic due to intermittent pulses of rainfall driven primary productivity (Noy‐Meir, 1973). Remote sensing provides the opportunity for changes in vegetation to be measured with high spatial and temporal resolution (Almalki et al., 2022). In drylands, this allows researchers to investigate how wild herbivores interact with variable vegetation dynamics more easily than traditional survey methods (e.g., Fisher et al., 2021; Velamazán et al., 2023).

Fractional vegetation cover measurements can effectively evaluate grazing impacts in dryland ecosystems (Retallack et al., 2024). Remotely sensed fractional photosynthetic (PV) and non‐photosynthetic (NPV) vegetation cover can represent the capacity of drylands to retain soil and water (Wei et al., 2023) and measure basal resource availability for living and detrital food webs (Dashpurev et al., 2023; Post, 2002). However, remotely sensed data are not commonly applied to experimental studies addressing wild herbivore impacts (Tai et al., 2021). Integrating remotely sensed vegetation measurements into existing experimental designs could bridge gaps with in situ studies to progress our understanding of herbivore‐vegetation interactions in spatially and temporally variable ecosystems (Hudson et al., 2020).

In dryland regions of southeastern Australia there is evidence that wild kangaroos *Macropus* and *Osphranter* sp. can at times overgraze conservation reserves where livestock have been removed (Mills et al., 2020; Prowse et al., 2019). Kangaroo populations periodically reach hyper‐abundances during sustained or successive green pulses (Lunney et al., 2018), particularly where dingo *Canis lupus dingo* suppression to facilitate sheep production has removed top‐down pressures on their populations (Pople et al., 2000). Under these circumstances, kangaroos may limit grass and forb cover, which can impact food availability for other herbivores and granivores (Finlayson et al., 2021; Rees et al., 2019). Intense kangaroo grazing during and after green pulses can also prevent senescent vegetation from accumulating, which can reduce the diversity and abundance of invertebrate detritivores and their predators (Wijas et al., 2022, 2023).

European rabbits *Oryctolagus cuniculus* also threaten vegetation recovery on dryland conservation reserves (Cooke, 2012). Rabbits first invaded Australia's arid rangelands in the late 1800s (Alves et al., 2022) causing extensive vegetation destruction and land degradation (Lunney, 2001). The release of the myxoma virus in 1950 resulted in a massive decline in rabbit populations (Fenner & Ratcliffe, 1965). Subsequent releases of the rabbit haemorrhagic disease virus (RHDV) from 1996 onwards along with intense localized active management practices have also suppressed rabbit populations so that they now occur at much lower numbers than they did before 1950 (Mutze et al., 2010). However, even at low to moderate densities rabbits can impact vegetation structure and cover by suppressing woody shrubs, forbs, and grasses (Braden et al., 2021; Leigh et al., 1989; Munro et al., 2009; Mutze et al., 2016a). Consequently, rabbits have been implicated in the endangerment of 322 native species listed as threatened under Australia's *Environment Protection and Biodiversity Conservation Act 1999* (EPBC Act, 1999).

Successful herbivore management for vegetation recovery relies on understanding spatial and temporal trends in herbivore impacts (Hudson et al., 2020). Since the release of RHDV1, some studies have suggested that rabbits continue to have stronger effects on vegetation composition and cover than kangaroos (Mutze et al., 2016b). Others have found that both herbivores limit grass cover and reduce total vegetation biomass to similar extents (Braden et al., 2021; Mills et al., 2020). These results may be contradictory because many of these studies had low temporal or spatial replication (Wang et al., 2024). Hence, they may have failed to capture temporal shifts in the dominance between rabbits and kangaroos under different management practices (Berman et al., 2011; Gordon et al., 2021), predator assemblages (Mills et al., 2021; Stobo‐Wilson et al., 2020), or habitat types (Cairns et al., 1991; Jansen et al., 2023). Determining whether the dominant herbivore limiting vegetation cover shifts through space, time, or both could greatly improve evidence‐based management of grazing pressure by giving land managers information they could use to prioritize their management of different herbivore species.

In response to concerns over grazing impacts from rabbits and kangaroos, experimental kangaroo exclusion, kangaroo plus rabbit exclusion, and control plots have been independently established on several conservation reserves throughout southeastern Australia's drylands. To investigate how either species affects vegetation cover, we use seasonal time series measurements of fractional PV and NPV cover from three reserves between winter 2016 and spring 2023 where experimental plots were large enough to collect Sentinel‐2 satellite imagery measurements. By comparing vegetation cover measurements within treatments, between reserves, and along temporal rainfall gradients, we test whether kangaroos or rabbits are the dominant species limiting vegetation cover on dryland reserves in southeastern Australia, whether this effect is consistent among reserves, and whether this effect varies with antecedental rainfall.

## METHODS

### Study sites

**FIGURE 1:**
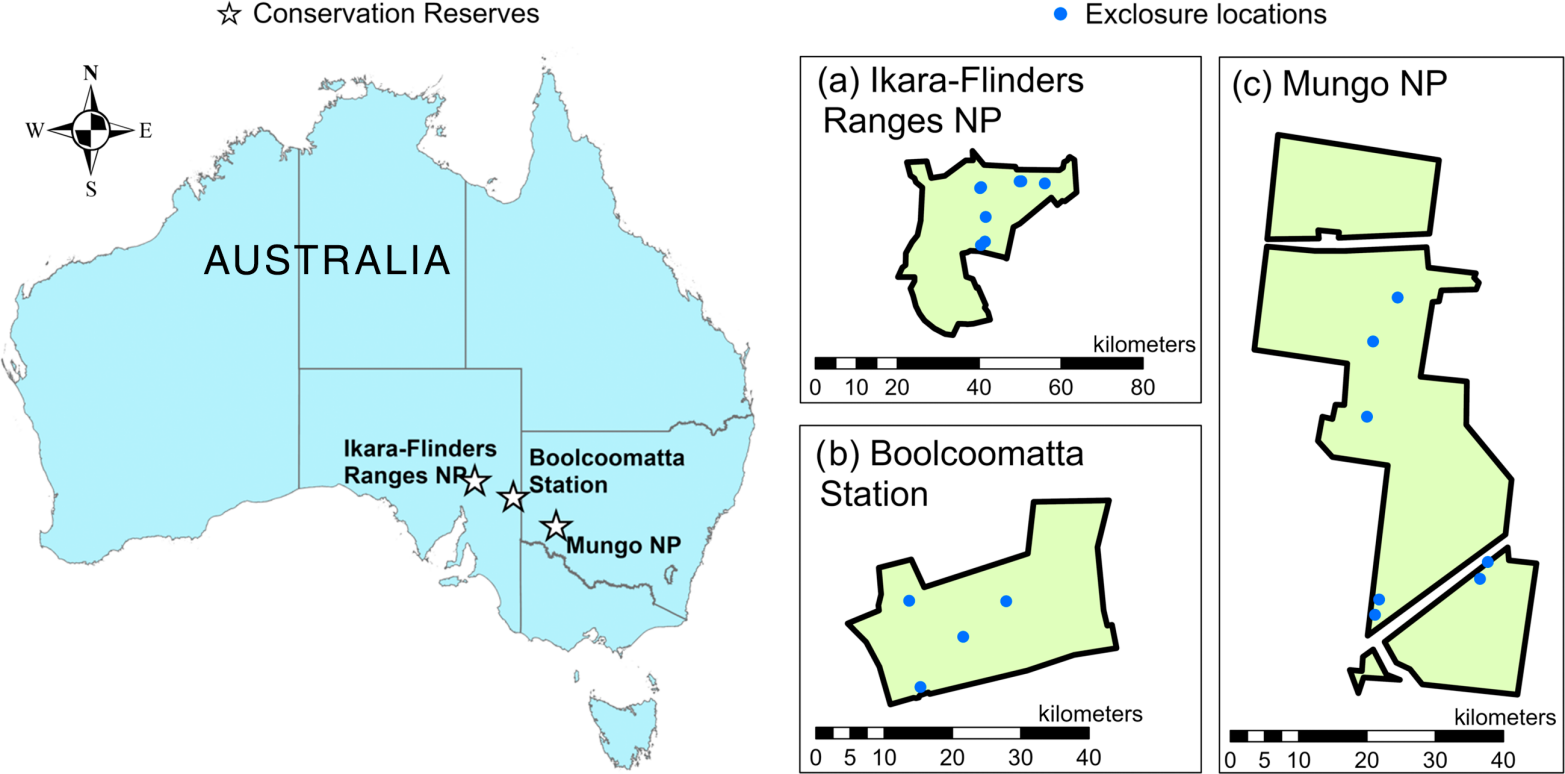
Map of study sites with locations of treatment sets in (a) the Ikara‐Flinders Ranges National Park, (b) Boolcoomatta Station, and (c) Mungo National Park.

Data were collected from Mungo National Park in New South Wales, and the Ikara‐Flinders Ranges National Park and Boolcoomatta Station in South Australia (Figure 1). Our sites are distributed along a dryland rainfall gradient with annual mean rainfall totals of 194 mm at Boolcoomatta, 280 mm at Mungo, and 307 mm in the Ikara‐Flinders Ranges. However, interannual rainfall can vary significantly at all three sites.

**FIGURE 2:**
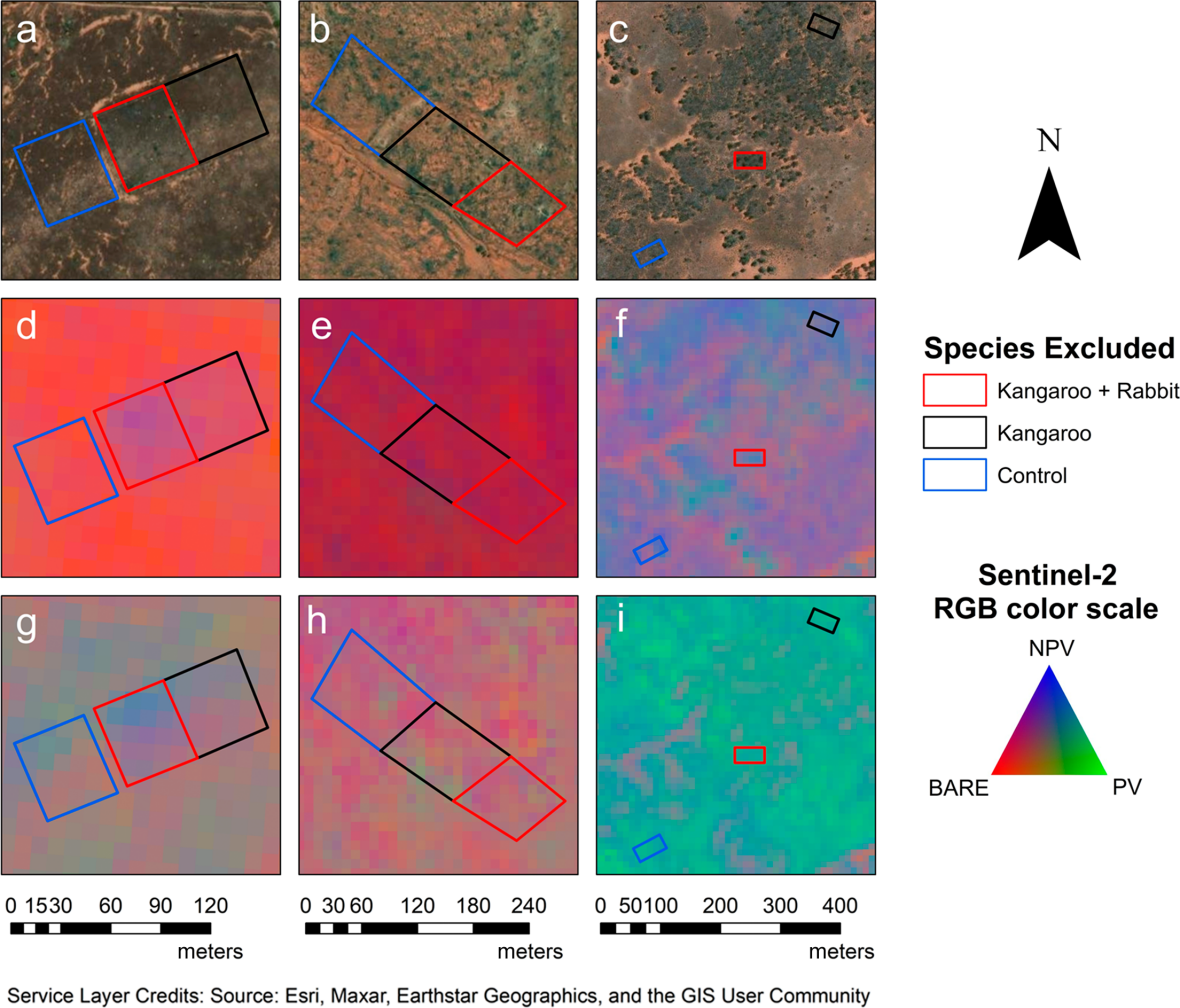
Examples of kangaroo + rabbit exclusion (red), kangaroo only exclusion (black) and control plots (blue) with satellite imagery at (a) Ikara‐Flinders Ranges NP, (b) Boolcoomatta, and (c) Mungo NP, Sentinel‐2 seasonal fractional cover during drought in summer 2020−2021 (d–f), and Sentinel‐2 seasonal fractional cover during a resource pulse in spring 2022 (g–i). Color scale relates to percentage of bare ground (Bare), photosynthetic vegetation (PV), and non‐photosynthetic vegetation (NPV).

Each site contains replicate sets of kangaroo + rabbit exclusion, kangaroo only exclusion, and control plots (Figures 1 and 2) with Boolcoomatta containing four sets, Mungo containing seven sets, and the Ikara‐Flinders Ranges containing eight sets. Rabbit only exclusion was not established on all reserves, hence we assume that any differences between kangaroo only and kangaroo + rabbit exclusion plots were due to the removal of grazing pressure from rabbits. A minimum plot size of 30 m^2^ was required to obtain sufficient Sentinel‐2 pixels for reliable measurements, with plots ranging between 34 and 132 m^2^. All treatment and control plots in each reserve were dominated by a mix of annual or perennial understory grasses, forbs, and shrubs. Each plot also contained sparse overstory or midstory species. The time since each treatment was established varied, with exclusion fencing being constructed at Boolcoomatta in 2010, Mungo between 2000 and 2008, and the Ikara‐Flinders Rangers in 1999 except for one set that was constructed in 1985. All exclosures were established once livestock were removed, with destocking occurring in 2006 at Boolcoomatta, 1978 at Mungo, and 1945 in the Ikara‐Flinders Ranges.

Since their declaration as conservation reserves, the dominant herbivores at each site have been red kangaroo and rabbits. Previous field studies show that kangaroos and rabbits each have marked effects on vegetation structure and composition at each of the reserves (Mills et al., 2020; Mutze et al., 2016a, 2016b; Wijas et al., 2023; Xu et al., 2023). Feral goats *Capra hircus* are present at all three reserves; however, goat densities in the study areas are considered negligible, as they prefer other habitat types and are subjected to population control (Mills et al., 2020; Mutze et al., 2016b).

### Data collection

Processed Sentinel‐2 fractional vegetation cover data were downloaded from the Terrestrial Ecosystem Research Network for Mungo and Boolcoomatta with masks applied for cloud, cloud shadow, topographic shadow, and water (Flood, 2017) applied using code developed by the Joint Remote Sensing Research Program (JRSRP, 2021). For the Ikara‐Flinders Ranges, Sentinel‐2 images downloaded from the European Space Agency as Level 1C version 02.04 (European Space Agency, 2021) were processed using the same method and code applied by the JRSRP (Scarth et al., 2022). Fractional vegetation cover measurements derived from these products were based on Landsat‐5 TM and Landsat‐7 models (Guerschman et al., 2015), using Sentinel‐2 bands adjusted to match Landsat bands (Flood, 2017). This included spectral unmixing models based on comprehensive sampling of overstory and understory cover in a variety of habitats (Muir et al., 2011) that achieve a root mean squared error (RMSE) of 11.2% and 16.2% for PV and NPV, respectively when 3 × 3 Landsat pixels were compared with 100 m diameter field plots (Guerschman et al., 2015). RMSE between 30‐m Landsat and averaged 10‐m Sentinel‐2 fractional cover from the same day were only 3% and 6% for PV and NPV, respectively (Flood, 2017). Seasonal composites were finally created by taking the multi‐dimensional equivalent of the median for at least three measures of fractional vegetation cover (FVC) in every season of each pixel (Flood, 2013). Seasonal PV and NPV cover averages were obtained at each site for every season beginning in summer 2016 and continuing until spring 2023 (Smith et al., 2026).

Monthly rainfall totals were obtained from the Bureau of Meteorology. For each site, rainfall totals were obtained for the nearest rainfall station. Where monthly totals were missing, a substitute value was taken from the next closest rainfall station to the respective study site. For each season, the rainfall total for the previous 12 and 24 months to the first month in each season was calculated. The total rainfall for the previous 12 months was used when analyzing PV because it has previously been demonstrated to have strong correlations with primary productivity in arid and semi‐arid Australia, while the rainfall for the previous 24 months was used when analyzing NPV cover because this period better accounts for the accumulation of senescent vegetation over time (Guerschman et al., 2020).

### Data analysis

Generalized linear mixed models (GLMMs) were run using the *glmmTMB* package (Brooks et al., 2017) in R version 4.3.1 to test the fixed effects of rainfall, herbivore exclusion, conservation reserve, and interactions on green and senescent cover. To account for additional variation (e.g., vegetation type, soil type, distance from rabbit warrens, distance from water) each pair of plots was set as a random factor along with the years since each plot was constructed. An autocorrelative 1 covariance term was also included to account for temporal autocorrelation between measurements from successive sampling periods within each conservation reserve. Tweedie and gaussian error distributions were used to model PV and NPV cover respectively. Both response variables were log transformed to improve Akaike information criterion (AIC) scores compared to untransformed (PV ΔAIC = 10643.14, NPV ΔAIC = 15249.01) and square root transformed models (PV ΔAIC = 4576.48, NPV ΔAIC = 2093.2), and model fit was assessed by consulting residual plots. For each measurement, the *p* values for species exclusion, the respective antecedental rainfall total, conservation reserve, and their interactions were retrieved by analysis of deviance tables with Type II Wald χ^2^ tests using the function ANOVA in the package *car*. *p* values less than 0.05 were considered significant and would indicate that any fixed factor or interaction significantly influenced the respective fractional cover measurement.

## RESULTS

### Trends in 12‐ and 24‐month antecedental rainfall patterns

**FIGURE 3:**
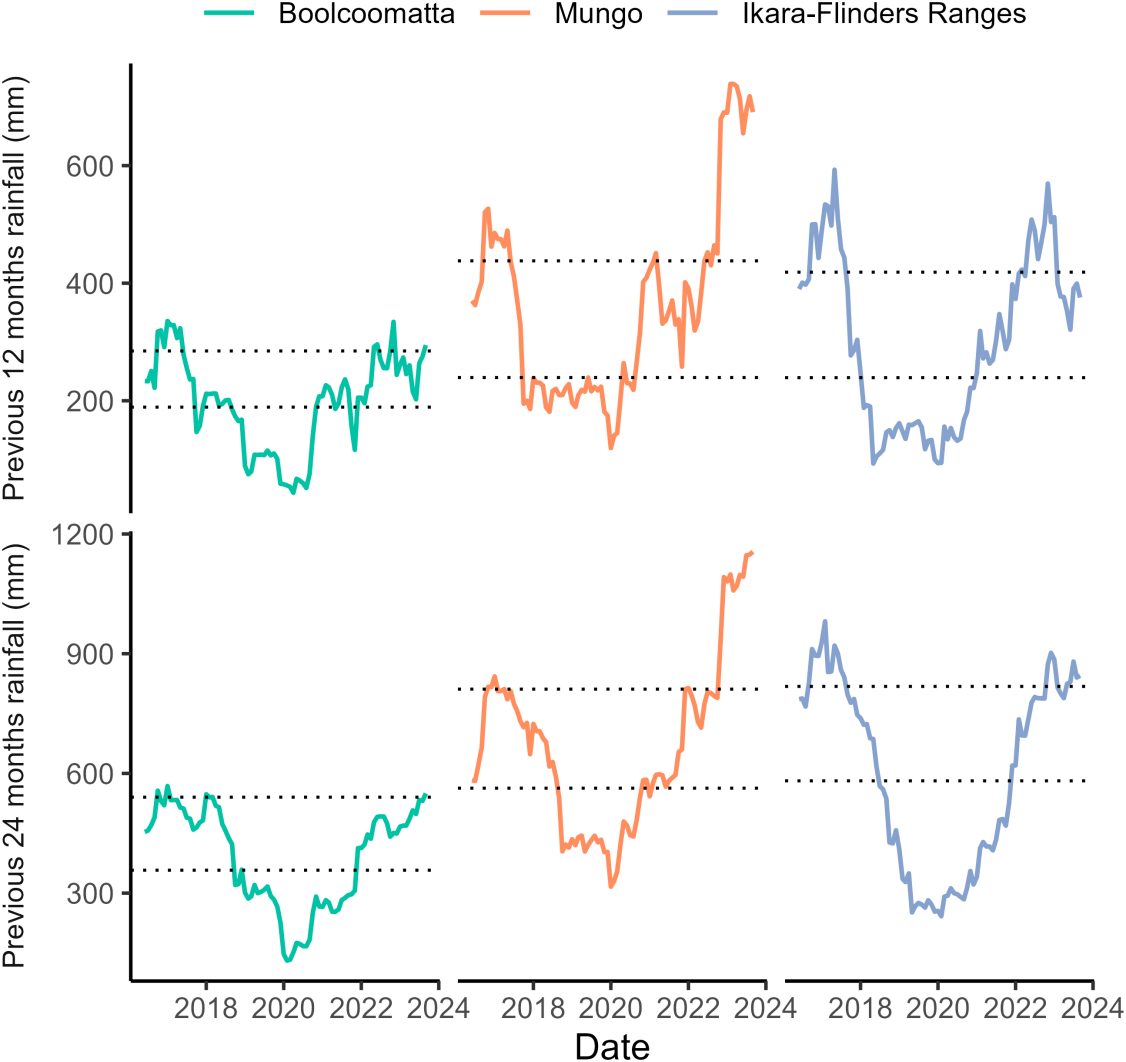
Time series of antecedental rainfall totals from the previous 12 months at (a) Boolcoomatta, (b) Mungo, and (c) Ikara‐Flinders Ranges and previous 24 months at (d) Boolcoomatta, (e) Mungo, and (f) Ikara‐Flinders Ranges with long term 25th and 75th percentile values (gray dotted line) for each location.

Temporal rainfall trends were similar at Boolcoomatta, Mungo, and the Ikara‐Flinders Ranges (Figure 3). Relative to the long‐term 75th percentile totals, rainfall was high in the 12 and 24 months prior to December 2016. Rainfall did however decline in 2017 and remained low until February 2020. An upwards trend in rainfall then persisted throughout the region until spring 2023 when the final FC measurements were retrieved. Throughout the study period, rainfall varied significantly both within and between sites. The lowest 12‐ and 24‐month rainfall totals of 53.5 and 133.0 mm were recorded at Boolcoomatta in March 2020, while the highest 12‐ and 24‐month totals of 739.0 and 1155.5 mm were recorded at Mungo in March and September 2023, respectively. During the study period, rainfall was on average lowest at Boolcoomatta (12 months = 195.4 mm, 24 months = 389.1 mm) and highest at Mungo (12 months = 363.9 mm, 24 months = 674.0 mm) despite Mungo having lower long term median values for 12 and 24 months than the Ikara‐Flinders Ranges.

### Fractional vegetation cover time series

**FIGURE 4:**
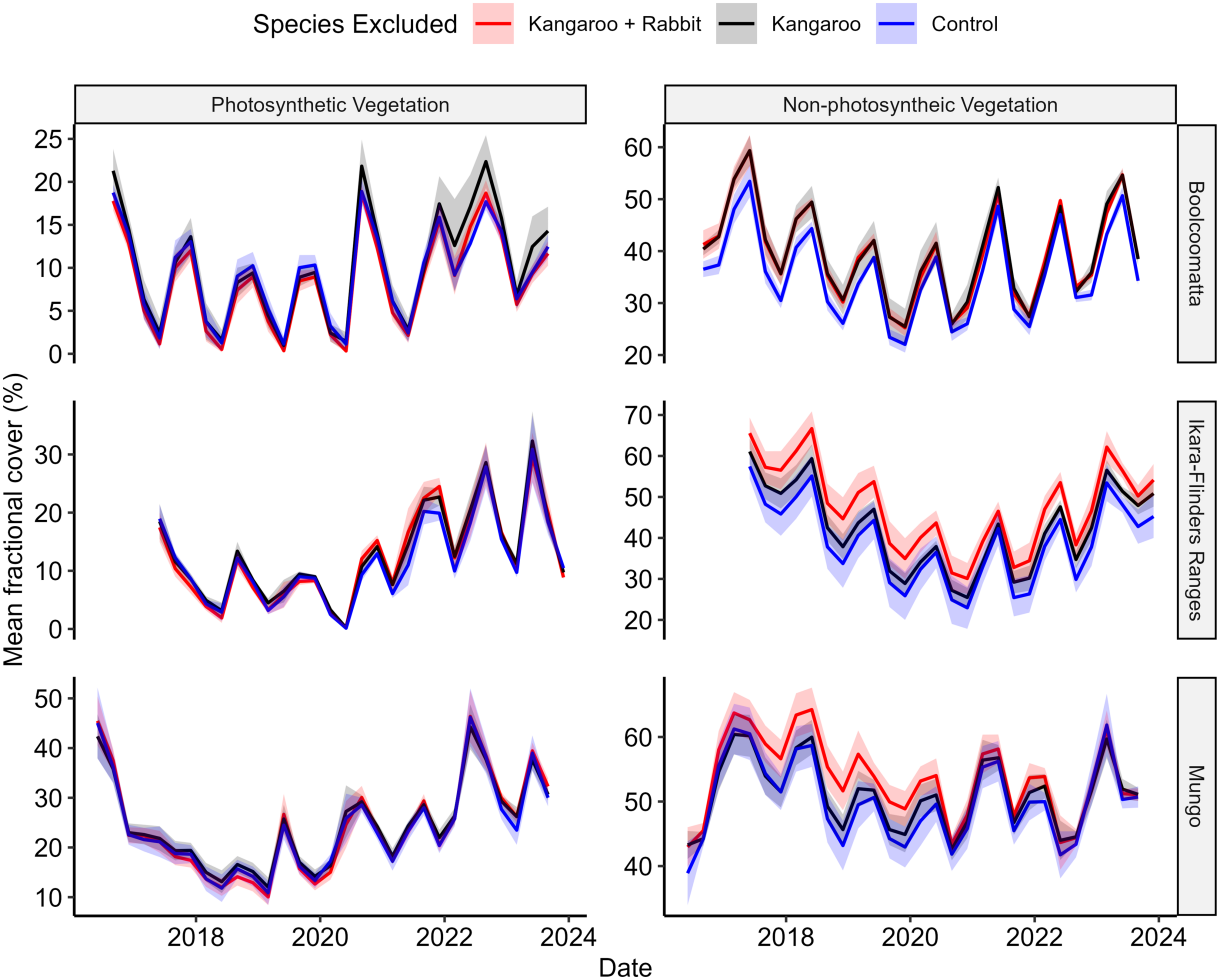
Time series of mean Sentinel‐2 percentage fractional photosynthetic vegetation and non‐photosynthetic vegetation cover measurements with one SE between winter 2016 and spring 2023 in kangaroo + rabbit exclusion (red), kangaroo only exclusion (black), and control plots (blue) at Boolcoomatta Station, The Ikara‐Flinders Ranges National Park, and Mungo National Park.

Sentinel‐2 fractional vegetation cover measurements were available between Winter 2016 and Spring 2023 at Mungo, Winter 2017 and Summer 2023 in the Ikara‐Flinders Ranges, and Spring 2016 to Spring 2023 at Boolcoomatta. Vegetation cover fluctuated seasonally with PV typically peaking in Spring or Summer in each reserve followed by peaks in NPV in Autumn or Winter (Figure 4). PV cover at Mungo did not follow this trend between winter 2016 and autumn 2019 however, as PV cover declined consistently during this period before resuming seasonal cycles.

### Effect of rainfall, grazing, and conservation reserve on vegetation cover

**FIGURE 5:**
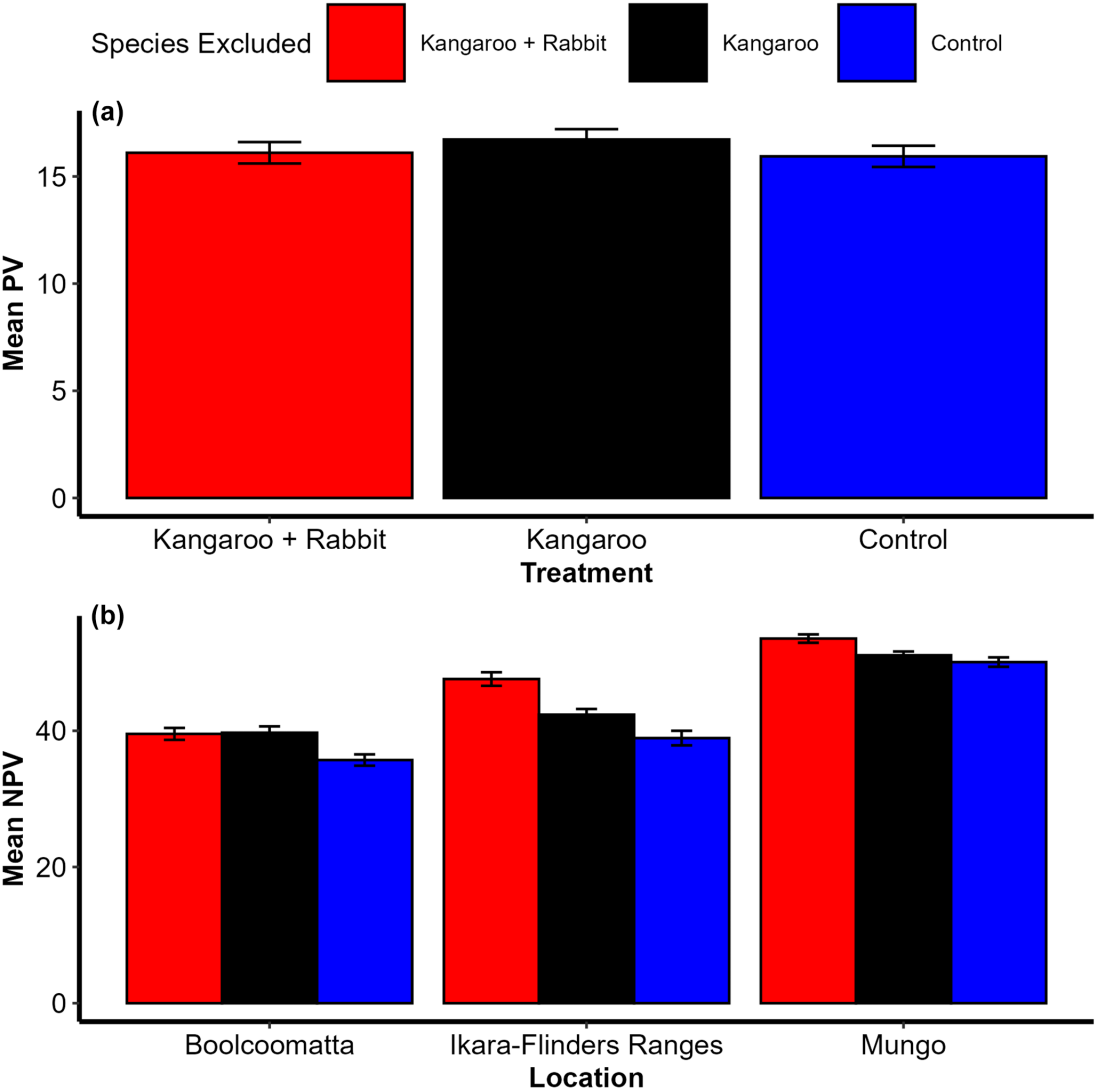
Bar plots of (a) mean percentage fractional photosynthetic vegetation (PV) cover and (b) percentage fractional non‐photosynthetic vegetation (NPV) cover with one SE bars at Boolcoomatta, Ikara‐Flinders Ranges, and Mungo in kangaroo + rabbit exclusion plots (red), kangaroo only exclusion plots (black) and control plots (blue).

The PV GLMM (*R* ^2^_*m*_ = 0.495, *R* ^2^_*c*_ = 0.862) showed PV cover responded positively to 12‐month antecedental rainfall at all sites (Table 1). PV cover also varied between reserves, being on average highest at Mungo and lowest at Boolcoomatta (Figure 4). PV cover was marginally higher in kangaroo‐only exclusion plots compared to control and kangaroo + rabbit exclusion plots; however, the difference between control and kangaroo + rabbit exclusion plots was not significant (Figure 5a).

**TABLE 1:** Generalized linear mixed model ANOVA output table for photosynthetic and non‐photosynthetic vegetation cover.

Response variable	Predictor variables	Chi‐square	DF	Pr(>Chi‐square)
Photosynthetic vegetation cover	Previous 12 months rainfall	17.1586	1	**0.0002**
	Species exclusion	13.0308	2	**6.0e** ^**−6**^
	Conservation reserve	24.7303	2	0.171
	Prev. 12 m rainfall × species exclusion	3.9960	2	0.242
	Prev. 12 m rainfall × conservation reserve	2.3103	2	0.411
	Species exclusion × conservation reserve	3.7035	4	0.217
	Prev. 12 m rainfall × species exclusion × conservation reserve	1.8377	4	0.701
Non‐photosynthetic vegetation cover	Prev. 24 m rainfall	12.5616	1	**0.0006**
	Species exclusion	224.9006	2	**<2.2e** ^**−16**^
	Conservation reserve	4.9417	2	0.190
	Prev. 24 m rainfall × species exclusion	10.3983	2	**0.0008**
	Prev. 24 m rainfall × conservation reserve	3.3909	2	0.534
	Species exclusion × conservation reserve	67.8434	4	**<2.2e** ^**−16**^
	Prev. 24 m rainfall × species exclusion × conservation reserve	1.9899	4	0.662

*Note*: Analysis of deviance uses the Wald type II Chi‐squared test. Bold indicates significant values (*p* < 0.05).

**FIGURE 6:**
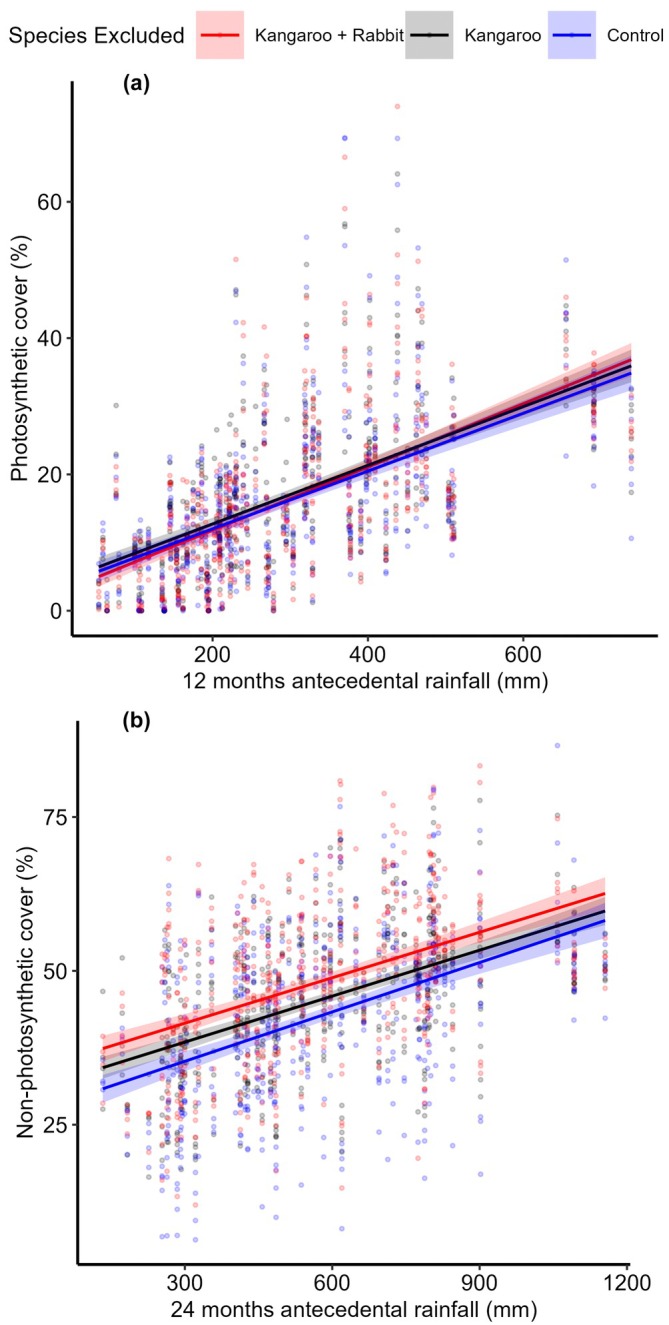
Scatter plot of (a) percentage photosynthetic vegetation cover and 12‐months antecedental rainfall and (b) percentage non‐photosynthesis vegetation cover and 24‐months antecedental rainfall with lines of best fit and SE ribbons for kangaroo + rabbit exclusion plots (red), kangaroo only exclusion plots (black), and control plots (blue).

The NPV GLMM (*R* ^2^_*m*_ = 0.239, *R* ^2^_*c*_ = 0.765) showed there was a significant interaction between the previous 24 months rainfall and species exclusion for NPV cover (Table 1). NPV cover at Boolcoomatta was significantly lower in control plots compared to the kangaroo exclusion and kangaroo + rabbit exclusion plots; however, there was no difference between kangaroo exclusion and kangaroo + rabbit exclusion. In the Ikara‐Flinders Ranges, NPV cover was significantly lower in control plots compared to kangaroo exclusion plots, lower in control plots compared to kangaroo + rabbit exclusion plots, and lower in kangaroo exclusion plots compared to kangaroo + rabbit exclusion plots. At Mungo, there was no significant difference between control plots or kangaroo exclusion plots, while NPV cover was significantly higher in the kangaroo + rabbit exclusion plots compared to both the control and kangaroo exclusion plots (Figure 5b). There was also a significant interaction between treatment and rainfall, with herbivore exclusion having a stronger effect on NPV as antecedental rainfall declined (Figure 6b).

## DISCUSSION

The impacts of kangaroo and rabbit grazing varied between each herbivore, conservation reserve, and between PV and NPV cover (Table 1). We found evidence that only kangaroo grazing limited PV cover and that this effect was relatively weak across all reserves (Figure 5a) and antecedental rainfall totals (Figure 6a). In contrast, grazing by both rabbits and kangaroos had stronger impacts on NPV cover. The impacts of either herbivore also varied spatially with evidence that NPV was limited by kangaroos only at Boolcoomatta, rabbits only at Mungo, and both rabbits and kangaroos in the Ikara‐Flinders Ranges (Figure 5b). Furthermore, both herbivores had stronger impacts on NPV cover as antecedental rainfall decreased (Figure 6b). Our results suggest that different factors may mediate biomass consumption by kangaroos and rabbits, and that spatial variability in these factors may determine which herbivore is the primary grazer threatening vegetation recovery at a reserve scale.

Only kangaroo grazing had a statistically significant albeit week effect on green cover. Primary productivity can exhibit marked temporal fluctuations in dryland systems as grasses and forbs respond rapidly during rainfall‐driven resource pulses before senescing as rainfall declines (Noy‐Meir, 1973). Both kangaroos and rabbits graze grasses and forbs (Dawson & Ellis, 1996), and either herbivore can reduce pasture cover and biomass in dryland grasslands and grassy woodlands (Braden et al., 2021; Leigh et al., 1989; Mills et al., 2020; Mutze et al., 2016b). This effect is rarely observed in PV; however, possibly because many grasses and forbs senesce before being grazed or because sampling occurs after ungrazed vegetation has senesced (Fisher et al., 2021). Due to the frequent repeated measurements, our data support claims that kangaroo and rabbit grazing may in fact have trivial impacts on green cover in dryland environments at densities captured during our study.

Grazing by kangaroos and rabbits had stronger effects on NPV cover despite the weak to negligible effects on PV. In dryland systems, vegetation biomass is often dominated by senescent material (Barnetson et al., 2017; Okin, 2010) that decays slowly under moisture limiting conditions (Fierer et al., 2009). Deficits in NPV from disturbances may then also accumulate through time resulting in stronger signals of grazing impacts compared to PV vegetation (Fisher et al., 2021). The treatment effects on senescent cover align with previous research that has found both kangaroos and rabbits limit dryland vegetation biomass and cover where they are sufficiently abundant (Braden et al., 2021; Leigh et al., 1989; Mills et al., 2020; Mutze et al., 2016b). This suggests that wild herbivore grazing in dryland systems is likely to have stronger indirect impacts on ecosystem components supported by NPV such as detrital food webs compared to those supported by PV (Wijas et al., 2022, 2023).

The stronger effect of kangaroo exclusion during low rainfall periods (Figure 6b) leads us to hypothesize that regional rainfall patterns likely drove reserve‐scale effects of kangaroo grazing. There is a complex interplay between soil moisture, vegetation growth, and the density and grazing impacts of kangaroo populations. During wet periods, kangaroo populations increase but may have only limited effects on vegetation structure and composition because plant growth is not limited by moisture availability. When soil moisture declines, kangaroo grazing impacts on vegetation cover become more marked as densities do not immediately decline and there is no plant growth to replace consumed biomass (Fisher et al., 2021). Hence, high rainfall at Mungo from 2022 onwards likely dampened kangaroo impacts by removing bottom‐up limitations on vegetation cover while kangaroo population growth lagged behind increases in primary productivity. This also explains why our reserve‐scale results contrast with studies showing that kangaroos suppress senescent biomass during drought at Mungo (Braden et al., 2021; Mills et al., 2020) but not after high rainfall in the Ikara‐Flinders Ranges (Mutze et al., 2016b). Such discrepancies likely reflect temporal variability in kangaroo densities relative to productivity that are undetectable in short‐term studies. We therefore propose that kangaroo disturbance patterns will align with regional rainfall variability over space and time (Cairns et al., 1991).

The effects of rabbit grazing on NPV showed different spatial patterns to kangaroos suggesting different mechanisms drove their impacts at the reserve scale. Rabbit grazing reduced NPV at Mungo and in the Ikara‐Flinders Ranges but not at Boolcoomatta (Figure 5b). Extensive warren destruction (ripping) is the most effective method of managing local rabbit populations over periods up to 10 years (Mcphee & Butler, 2010). At Boolcoomatta, reserve‐wide warren ripping in 2012 significantly reduced rabbit populations (Mills et al., 2020) while severe drought in 2018–2019 likely prevented recolonisation (Cooke, 1982). In contrast, warren ripping at Mungo and the Ikara‐Flinders Ranges since 2002 has been less successful due to reduced predation pressure following successful fox *Vulpes vulpes* control (Stobo‐Wilson et al., 2020), or inadequate funding (WLLS, 2020). While biological control agents have historically reduced rabbit populations throughout the study region (Mutze et al., 2010), continued comprehensive on‐ground management is likely required to prevent recolonisation from nearby active warrens (Ramsey et al., 2014) and minimize local impacts of rabbit grazing.

Different habitat preferences may have also driven different spatial patterns of grazing impacts from rabbits and kangaroos. Rabbit warrens in arid systems are typically found in sandy loams, dunes, or creek lines with soft soil and dense vegetation nearby open areas with grasses and forbs for foraging (Jansen et al., 2023; Moseby et al., 2005). Rabbit grazing is most intense in these habitats within 50 m of active warrens, declining rapidly thereafter (Leigh et al., 1989). In contrast, red kangaroos graze broadly through open grasslands and shrublands, with rainfall having a stronger influence on local densities than habitat type (Cairns et al., 1991). Rabbit impacts recorded at each reserve may not have been representative of entire properties depending on the proximity of sampled areas to suitable habitat and active burrows, while kangaroo densities were less likely influenced by habitat variability, making it unlikely that site‐scale kangaroo grazing impacts were driven by population differences within reserves. However, a limitation of our study is that we do not have data on the distances of each plot to suitable rabbit habitat or local rabbit densities that could reduce uncertainty surrounding drivers of spatial variability in rabbit grazing impacts.

A key finding from our study relevant for biodiversity conservation is the interactive effect of wild herbivore grazing and rainfall on NPV. By applying remotely sensed seasonal fractional cover measures over a 7‐year period to experimental exclusion plots, we were able to demonstrate that biomass consumption by wild herbivores can increase relative to available pasture as productivity decreases. In dryland ecosystems with pulse dynamics, NPV often dominates during periods of low productivity due to slow decomposition rates (Fierer et al., 2009). During these periods, faunal communities rely on brown food webs where energy and nutrients flow from senescent vegetation to detritivores and higher order species (Letnic & Dickman, 2010). Our data show that wild herbivores can prevent NPV from accumulating (Wijas et al., 2023), and that these grazing effects are strongest during periods of low rainfall.

A caveat of our study is that detailed responses among different vegetation types cannot be interpreted from remotely sensed satellite imagery that captures only the dynamics of FVC through space and time (Prabhakara et al., 2015). For example, PV was lower in plots excluding both rabbits and kangaroos than compared to kangaroo only exclusion plots despite experiencing less grazing pressure. Rabbit exclusion typically promotes higher shrub cover (Leigh et al., 1989; Mutze et al., 2016a; Mills et al., 2020) which can impact resource availability for understory species (Valladares et al., 2016). However, understory species can also benefit when shrubs provide shelter from grazing (Rahmanian et al., 2021) and enhanced ecosystem services such as improved soil quality (Rolo et al., 2012). Where rabbits and kangaroos were excluded, grasses and forbs may have been outcompeted by shrubs. Alternatively, reflectance data obtained by Sentinel‐2 sensors may not have captured understory cover beneath shrub canopies that contain more NPV material. This highlights that although remote sensing provides a powerful tool to measure general vegetation dynamics through space and time in experimental designs, in situ measurements are still required to understand nuanced phenological responses to different treatments.

Our results suggest that the impacts of sympatric wild herbivores can vary spatially with different factors likely driving the impacts of different herbivores at a local scale. We found evidence that the impact of kangaroo grazing decreased along spatial and temporal rainfall gradients, suggesting that kangaroo grazing is most likely to impact biodiversity during drought and in drier ecosystems. Although rabbit grazing also increased as antecedental rainfall decreased, spatial patterning in the impacts of rabbits was likely also driven by habitat characteristics or local management efforts that influence local population densities.

Our findings indicate that the timing of management interventions may be important in reducing grazing impact from wild herbivores. Insufficient herbivore management actions undertaken during resource pulses when grazing impacts are less likely to be observed may result in unsustainable grazing pressure from wild herbivores during subsequent low rainfall periods. However, highly mobile herbivores such as kangaroos operate at spatial scales beyond those tested in this study. Land managers would further benefit from modeling that predicts how wild herbivore populations respond at scales relevant to management actions. This would improve cost–benefit assessments of wild herbivore management actions made during high rainfall periods compared to low rainfall periods. Long‐term time series data should also be prioritized to better understand how precipitation, primary productivity, and wild herbivore population dynamics interact to impact flora and fauna. Addressing these knowledge gaps will allow for better evaluation of outcomes following control programs for both native and invasive herbivores and improve our understanding of when and where overgrazing by wild herbivores is likely to reduce vegetation cover and threaten biodiversity conservation outcomes.

## AUTHOR CONTRIBUTIONS

Matt Smith, Adrian Fisher, and Mike Letnic conceived the ideas and designed methodology. Matt S. Smith and Adrian G. Fisher collected the data. All authors contributed to analyzing and interpreting the data. Matt S. Smith led the writing of the manuscript and all authors contributed critically to the drafts and gave final approval for publication.

## CONFLICT OF INTEREST STATEMENT

The authors declare no conflicts of interest.

## Data Availability

The data used for this analysis and to generate figures are available from Smith et al. (2026) in Dryad at https://doi.org/10.5061/dryad.j0zpc86rb. Where available, fractional vegetation cover estimates were obtained from pre‐processed Sentinel‐2 data (Joint Remote Sensing Research Program & Department of the Environment, 2021) at https://portal.tern.org.au/metadata/30aa6403-5efb-47cd-bbbb-652d5c865df8. Otherwise, publicly available raw Sentinel‐2 data were obtained from the European Space Agency (Copernicus Sentinel‐2, processed by the European Space Agency, 2021; https://doi.org/10.5270/S2_-742ikth).
